# Large-Scale Proteomics Differentiates Cholesteatoma from Surrounding Tissues and Identifies Novel Proteins Related to the Pathogenesis

**DOI:** 10.1371/journal.pone.0104103

**Published:** 2014-08-05

**Authors:** Anders Britze, Rune Isak Dupont Birkler, Niels Gregersen, Therese Ovesen, Johan Palmfeldt

**Affiliations:** 1 Department of Otorhinolaryngology, Head and Neck Surgery, Aarhus University Hospital, Aarhus, Denmark; 2 Research Unit for Molecular Medicine, Aarhus University Hospital, Aarhus, Denmark; Deutsches Krebsforschungszentrum, Germany

## Abstract

Cholesteatoma is the growth of keratinizing squamous epithelium in the middle ear. It is associated with severe complications and has a poorly understood etiopathogenesis. Here, we present the results from extensive bioinformatics analyses of the first large-scale proteomic investigation of cholesteatoma. The purpose of this study was to take an unbiased approach to identifying alterations in protein expression and in biological processes, in order to explain the characteristic phenotype of this skin-derived tumor. Five different human tissue types (cholesteatoma, neck of cholesteatoma, tympanic membrane, external auditory canal skin, and middle ear mucosa) were analyzed. More than 2,400 unique proteins were identified using nanoLC-MS/MS based proteomics (data deposited to the ProteomeXchange), and 295 proteins were found to be differentially regulated in cholesteatoma. Validation analyses were performed by SRM mass spectrometry. Proteins found to be up- or down-regulated in cholesteatoma were analyzed using Ingenuity Pathway Analysis and clustered into functional groups, for which activation state and associations to disease processes were predicted. Cholesteatoma contained high levels of pro-inflammatory S100 proteins, such as S100A7A and S100A7. Several proteases, such as ELANE, were up-regulated, whereas extracellular matrix proteins, such as COL18A1 and NID2, were under-represented. This may lead to alterations in integrity and differentiation of the tissue (as suggested by the up-regulation of KRT4 in the cholesteatoma). The presented data on the differential protein composition in cholesteatoma corroborate previous studies, highlight novel protein functionalities involved in the pathogenesis, and identify new areas for targeted research that hold therapeutic potential for the disease.

## Introduction

Cholesteatoma is a tumorous growth of keratinizing squamous epithelia in the middle ear that is reported to affect around 7–9 people per 100.000/year in Europe [1], [2]. The observed expansive, destructive, and invasive characteristics share similarities with malignant diseases, which is mirrored by the extensive surgery and control regimens. Most patients experience severe complications ranging from hearing loss to potentially fatal intracranial infections, and there is a pressing need for developing medical treatment alternatives, based on the molecular pathology.

The basis for the development of cholesteatoma is ectopic keratinizing epithelial cells in the middle ear cavity, but its etiopathogenesis is not fully understood. The introduction of keratinizing epithelia to the middle ear is thought to arise mainly from retraction-pockets and/or thinning of the tympanic membrane, which are prevalent conditions in middle ear pathology, but may also stem from the immigration of cells through tympanic membrane perforations, metaplasia of the middle ear keratinocytes, migration of external auditory canal (EAC) keratinocytes, and embryonic remnants [3]. The presence of keratinocytes in the tympanic cavity alone, however, does not necessarily lead to cholesteatoma formation, which is supported by the finding of low number of cholesteatoma formations from tympanic membrane perforations, and the varying success rates of simple animal skin graft models [4], [5]. Accordingly, additional unknown factors are involved in the pathogenesis of cholesteatoma. Various molecular factors, such as differentiation [6]–[12], growth/proliferation [13]–[17], apoptosis [18], [19], inflammation [20], infection [21], [22] bone erosion, lipid metabolism [23] [24] and angiogenesis [25], with possible roles in the development and behavior of cholesteatoma have been investigated [26]. The majority of studies have focused only on one or a few markers for the areas of interest, and due to differences in study design results are not easily compared between these studies. Inflammation and bone erosion are frequent observations, but the promoting molecular processes behind are not fully understood. The roles of other biological processes in cholesteatoma remain controversial.

Technological development has greatly enhanced untargeted OMICS-methods in transcriptomics and proteomics. These methods can produce larger and more coherent analyses compared to the traditional targeted analyses and aid the development of new hypotheses. In cholesteatoma research, large data sets have been produced by transcriptomics using microarrays [27]–[30]. mRNA analyses are important for the process of explaining the biology, but the interpretation of results has to be careful due to the very low concordance (around 20%) with protein expression [31]. Some of the most consistent findings from mRNA studies revealed up-regulated transcripts for a number of proteins of the S100 group [27], [29], [30]; many of the S100 proteins have strong pro-inflammatory capabilities, but their role and protein expression in cholesteatoma is not known. New approaches must be utilized in order to achieve a greater understanding of the etiopathogenesis, connect the results of earlier studies, and correlate the transcriptional profile to the protein expression.

This study is the first to employ large-scale proteomics with subsequent bioinformatics in the investigation of cholesteatoma. The purpose was to compare the proteomes of cholesteatoma and its surrounding tissues, thereby taking an unbiased approach to identify biological functions and pathways involved in the pathogenesis of the disease. The inclusion of five distinct tissue types in the analysis (cholesteatoma, neck of cholesteatoma, tympanic membrane, external auditory canal skin, and middle ear mucosa), instead of the usual two (cholesteatoma and skin), led to the identification of protein-levels unique for these tissues. Bioinformatics analyses were performed stringently on the most highly-regulated proteins in cholesteatoma. The strongest results from these analyses describe reductions/degradations of extracellular matrix/basement membrane proteins, possibly caused by inflammation induced proteolysis, which may have widespread consequences for the integrity, differentiation and survival of the tissue. This new overview of expression patterns and suggested disease mechanisms may improve the understanding of the disease and create a base for targeted analyses that focus on possible drug targets, such as inflammatory mediators or proteases.

## Materials and Methods

### Ethics Statement

Tissue samples were collected by senior surgeons from patients undergoing cholesteatoma surgery at the department for Oto-Rhino-Laryngology Head and Neck Surgery at the Aarhus University Hospital in Denmark. Informed written consent was obtained from adult patients and from parents or legal guardians on behalf of minors and children prior to surgery. All procedures were carried out in compliance with the principles of the Declaration of Helsinki (1964) and with the permission from The Danish Research Ethics Committee (M-20090142). Data handling procedures were approved by the Danish Data Protection Agency (2010-41-4378).

### Patients and samples

For the large-scale analyses nine patients were included (five males, four females; age range: 7–77 years). Five biopsies were taken from each patient (same side): 1) Cholesteatoma sack, 2) Neck of cholesteatoma (the transition zone from the tympanic membrane), 3) Tympanic membrane (location remote from transition zone), 4) External auditory canal skin (deep part, few millimeters from the annulus), and 5) Middle ear mucosa (promontory area). The five tissues within a sample set were compared to each other such that each patient was his or her own control; this removed the effect of inter-individual differences, specifically, the differences in timing and inflammation state of the disease, and made it possible to follow the development in protein expression from apparent normal ear canal skin to pathologic cholesteatoma tissue and to evaluate the differences between neighboring tissues. Exclusion criteria: Unclear anatomy (no clear transition from the tympanic membrane to the cholesteatoma sack), use of topic/systemic corticosteroids within 2 weeks prior to surgery, and ongoing exudative/suppurative inflammation/infection at the time of surgery. Patients had not used antibiotics within two weeks prior to surgery.

### Sample preparation and pre-fractionation

**Figure 1:**
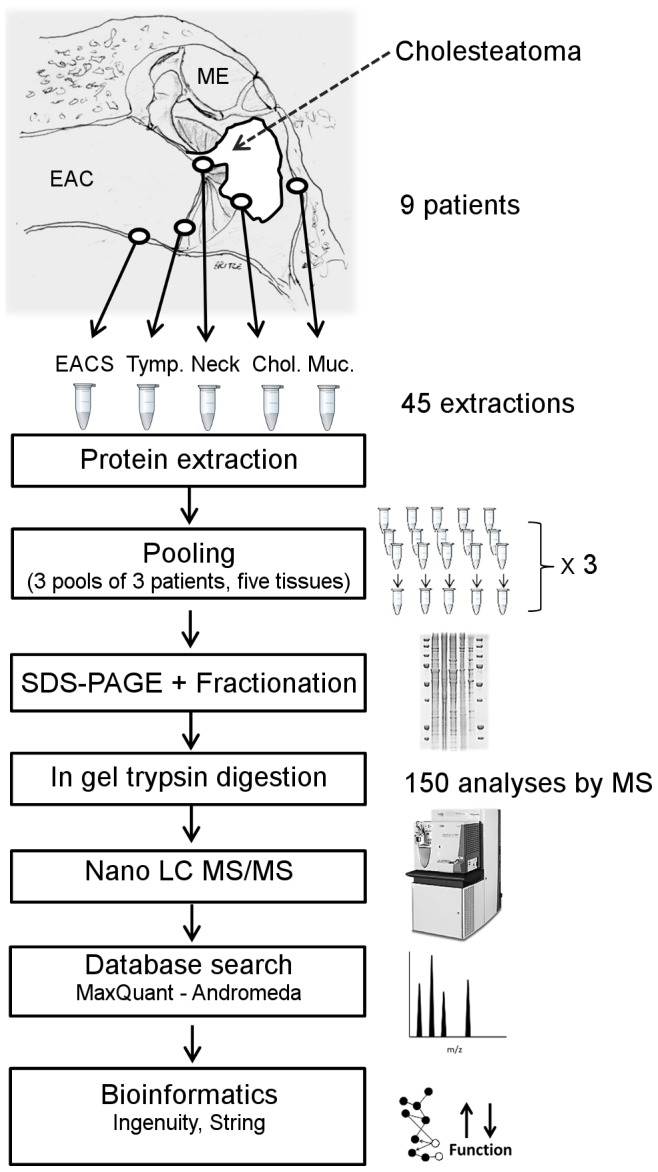
Simple overview of workflow. EACS: external auditory canal skin; Tymp: tympanic membrane; Neck: neck of cholesteatoma; Chol: cholesteatoma; Muc: middle ear mucosa; ME: Middle ear; LC: Liquid Chromatography; MS: Mass Spectrometry.

All samples were snap frozen in liquid nitrogen and stored at −80°C until further processing. Samples were minced with a scalpel on ice block and transferred to lysis/extraction buffer containing T-PER buffer (PIERCE), SDS 2%, DTT 30 mmol/l added fresh, glycerol 10%, HALT proteinase inhibitor cocktail including EDTA (PIERCE), and immediately put on a heat shaker at 70°C for 15 min. After that, samples underwent further homogenization using a Micro Grinding Kit (GE Healthcare) on ice for 1.5 minutes, followed by the addition of antifoam Y-30 1% (Sigma), sonication on ice (Branson Sonifier 250), and centrifugation at 20,000 g for 10 min, 4°C. Protein quantification of supernatant by Protein DotMetric (G-Biosciences) and precipitation/up-concentration by SDS-PAGE Clean-Up kit (GE Healthcare) were performed prior to SDS-PAGE. Equal amounts of protein from three different patient samples were pooled to get a sufficient total protein amount (>30 µg), resulting in three sample sets (five tissues), each comprising three pooled patient samples (Figure 1). GeLC-MS/MS methodology was then performed as previously described [32]. Briefly,the proteins were pre-fractionated by SDS-PAGE and digested by trypsin to prepare peptides for LC-MS/MS (Methods S1).

### LC-MS/MS and database searches

The three pools of samples were run in three separate mass spectrometry experiments. Each experiment comprised 50 runs (five tissue types, each split in ten fractions).

The peptide mixtures were separated by nano-liquid chromatography (Easy nLC from Proxeon, Odense, Denmark) coupled to mass spectrometry (LTQ-Orbitrap, Thermo Fisher Scientific, Bremen, Germany) through a nano-electrospray source with stainless steel emitter (Proxeon, Odense, Denmark). The peptides were separated on a reverse phase column, 75 µm in diameter and 100 mm long, packed with 3.5 µm Kromasil C18 particles (Eka Chemicals, Bohus, Sweden) at a flow rate of 300 nL/minute using a 100 min gradient of acetonitrile in 0.4% acetic acid, starting with 5% and ending with 35% acetonitrile. The mass spectrometry detection was full scan (m/z 400–2000) with Orbitrap detection at resolution r = 60,000 (at m/z 400), followed by up to four data-dependent MS/MS scans, with linear ion trap (LTQ) detection of the most intense ions. Dynamic exclusion of 25 s was employed as well as rejection of charge state +1.

Raw MS files were analyzed using MaxQuant (version 1.2.2.5) for protein identification and label-free quantification by means of peptide peak areas [33]. For protein identification the MS/MS spectra were searched using the Andromeda search engine [34] against the human Uniprot database [35]; release 2012_02 containing 20,255 reviewed protein sequences. In the main Andromeda search, precursor mass and fragment mass were searched with the initial mass tolerance of 12 ppm and 0.5 Da, respectively. The search included variable modifications of methionine oxidation, N-terminal acetylation, and fixed modification of carbamidomethylated cysteines. Minimal peptide length was set to 6 amino acids and a maximum of two missed trypsin cleavages was accepted. The false discovery rate (FDR) was set to 0.005 and 0.002 for peptide and for protein identifications, respectively. Peptides shared between two proteins were combined and reported as one protein group. Identifications from the reverse database were used only to estimate FDR of identification. Tables of identified proteins with quantitative data can be found in Tables S2 and S3. The mass spectrometry data from this publication, including RAW files and peptide and protein identifications, have been stored on the ProteomeXchange Consortium (http://proteomecentral.proteomexchange.org) via the PRIDE partner repository [36]; with the ProteomeXchange accession: PXD000457.

### Data treatment

The protein concentrations of the five tissue types were equilibrated before mass spectrometry (MS) and normalization of the mass spectrometry-measured intensities was performed, so that summed MS intensity for each sample was set to be equal.

Proteins that only were identified in one of the three experiments were excluded from further analysis. Differential expression (fold change) of the unique proteins within each experiment was assessed by natural logarithm transformation of the ratios. For cluster analysis and for overview distribution charts, the level in a tissue type was divided by the average level across the five tissue types. For the estimation of differential protein levels, ratios from pairwise tissue comparisons were used, as described below.

Two groups of differential level proteins were established based on the pairwise tissue comparisons: Group A: Proteins that were identified in all three experiments and showed two-tailed student's *t*-test *p* values <0.05 and a mean fold change >2× the standard error of the mean (SEM) based on a global standard error that was calculated from all quantified proteins within a pairwise comparison; this combination of *t*-test and fold change criteria was applied to isolate the stronger differential level estimates and has been used in our previous work [37]; Group B: Proteins that were identified in two or three experiments with larger variations in fold changes but with every single fold change (every single replicate) greater than 2× the SEM. Only proteins that did not meet the criteria for group A were tested against the criteria for group B. Analyzing the protein level differences in the comparisons that included cholesteatoma, the observed minimum fold changes of proteins meeting the criteria ranged from 2.7–7.9 (depending on the variation within the specific tissue comparison; Table 1).

**Table 1:** Minimum fold changes of proteins meeting the group A and B criteria for fold change.

Cholesteatoma vs.		Neck	Tymp	EACS	Mucosa
Fold change thresholds	Group A	7.9	3.1	2.7	2.6
	Group B	6.2	3.8	3	3.8

Only comparisons including cholesteatoma are shown. Neck: Neck of cholesteatoma; Tymp: Tympanic membrane; EACS: external auditory canal skin; Mucosa: Middle ear mucosa.

The assumption of normality of the logarithmically-transformed ratios was checked using qq-plots of the residuals. In order to calculate ratios in cases of intensity values below the lower limit of detection, zero-intensity values were exchanged with a value of three times the lowest measured intensity value of the dataset. The systematic presentation of data focused on cholesteatoma tissue and on the comparison of this with the other three keratinizing epithelia. Hierarchical clustering, based on the correlations of expression profiles of the five tissues (Pearson's correlation; average linkage) was performed. For this overview of the relation of the tissues, no criteria for fold changes were applied. Cluster 3.0 (http://bonsai.hgc.jp/~mdehoon/software/cluster) and Java TreeView (http://jtreeview.sourceforge.net/) software were used to analyze and display the data.

### Bioinformatics

For initial comparison of the protein identifications from the five tissues, pie-chart diagrams of the “biological processes” of the identified proteins in each tissue were produced using “Panther” classification system, ver. 7.2 (http://www.pantherdb.org). For more extensive pathway analyses Ingenuity Pathway Analysis (IPA), Ingenuity Systems, (www.ingenuity.com) was applied. Data sets from the pairwise tissue comparisons were uploaded to and analyzed by Ingenuity Pathway Analysis (IPA). Filters were set to only compare the proteins meeting the fold change criteria. The IPA analysis consisted of two parts: First, a functional analysis, where the sets of proteins with differentially altered levels from the tissue comparisons were associated with known canonical pathways and literature-based biological functions contained in the IPA Knowledge Base; see Methods S1 for details. In the second part of the IPA analysis, networks of proteins with differentially altered protein levels were algorithmically synthesized based on the connectivity of the proteins. The top scoring network (based on the quality and number of connections) was further explored in STRING 9.0, (http://string-db.org) after addition of proteins with differentially altered levels associated with the Gene Ontology, (http://www.geneontology.org), GO term “basement membrane”.

The gene names used to denote the proteins refer to the HGNC nomenclature. http://www.genenames.org/

### Validation

For the validation by selected reaction monitoring (SRM) mass spectrometry, samples from ten patients were acquired (three of these patients were also included in the large-scale analysis). Samples were prepared as described above but were analyzed separately (not pooled), and only cholesteatoma and EACS tissues were compared. For details see Methods S1.

## Results

A proteomics discovery study was performed to map differences in protein composition between cholesteatoma and neighboring ear tissues (Figure 1). The processing of the different tissue types were performed under the same conditions in parallel throughout the experimental procedures from SDS gel pre-fractionation (Figure S1) to MS analysis.

In summary, our data handling consisted of: 1) protein identification from the peptides identified by mass spectrometry, 2) selection of consistently detected proteins for overview tissue comparisons, and 3) selection of proteins showing marked protein level differences between the tissues for bioinformatics analyses.

### Protein identification

**Figure 2:**
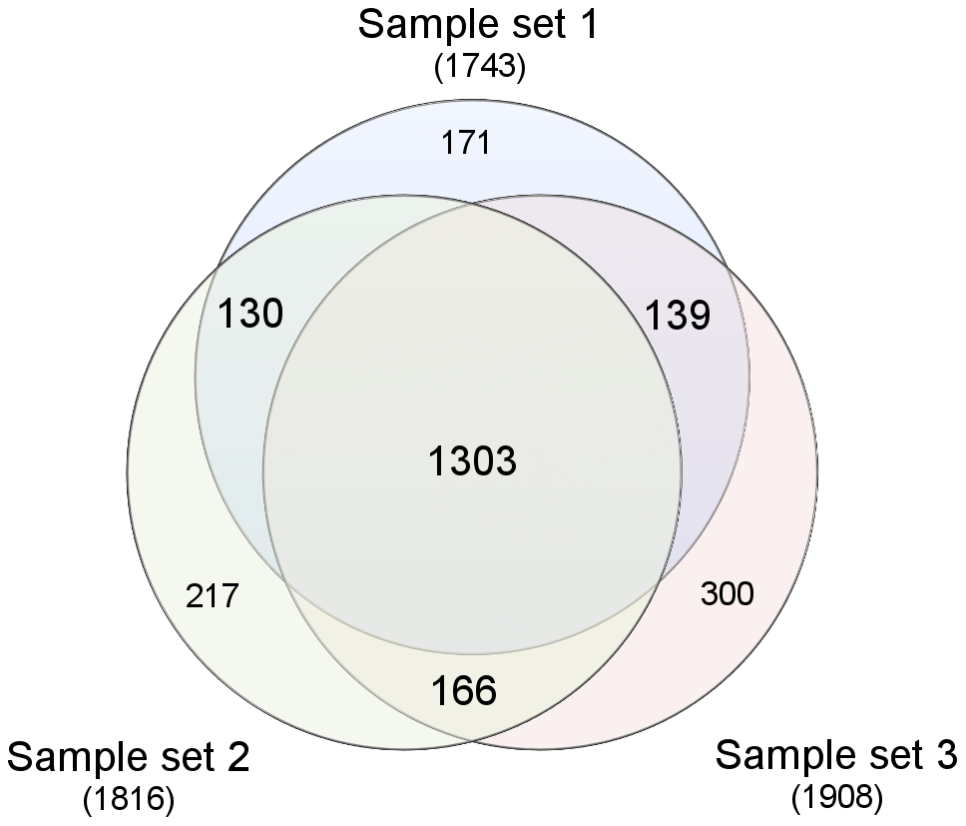
Identified proteins. Number and overlap of unique proteins identified in the three separate mass spectrometry experiments. In each experiment, a sample set of the five tissue types (pooled samples from three patients) were analyzed.

The three sample sets, each comprising five tissue types (Figure 1), were analyzed in three separate mass spectrometry experiments. More than 1700 proteins (False discovery rate <0.002) were identified, and relatively quantified, in each of the three experiments (Figure 2). Merging of the experiments yielded a total of 2426 unique proteins with 72% (1738) shared by at least two of the three experiments and 54% (1303) shared by all three experiments.

### Comparison of the protein profiles

The cluster analysis reflected the anatomical relationship between the tissue types (Figure S2). Among the four keratinizing epithelia, EACS was the most “distant relative”, suggesting a gradual shift in protein expression pattern from EACS through tympanic membrane and neck of cholesteatoma to cholesteatoma sack, and spoke against the possibility of cholesteatoma keratinocytes stemming directly from EACS. A comparison of pie-chart overviews of the protein profiles of the five tissues was performed based on the Gene Ontology categorization “biological process” (Figure S3). Some of the biological processes (e.g. 'metabolic process' and 'cellular process') were particularly well – represented. Altogether, the five tissues showed highly similar distributions.

### Pairwise tissue comparisons

Comparison of cholesteatoma with the four tissues (neck of cholesteatoma, tympanic membrane, EACS and mucosa) revealed that 26, 73, 159, and 153 proteins passed the filtering by *p* value and fold change and were included in the bioinformatics analyses (see Methods S1 and Table S1).

### Bioinformatics analysis

Instead of basing the analysis on only one or a few function-specific markers, the Ingenuity pathway analysis (IPA) performed large-scale literature-based associations of all proteins that met the fold change criteria (Protein groups A and B). pairwise For any given tissue comparison, less than seven percent of all proteins passed these criteria. This amount of pre-filtered proteins can yield strong biological associations while maintaining a high stringency. The top scoring 'biological functions', 'canonical pathways', and 'protein networks' that were specific for cholesteatoma tissue were compiled and summarized.

#### Biological functions

The IPA biological functions analysis calculates the probability of the sets of differentially expressed proteins being associated with known biological functions by chance alone. Furthermore, depending on the data, IPA has the capability to estimate whether biological functions are increased or decreased (see Materials and Methods). In many studies, the observed expression patterns within pathways are too chaotic for the estimation of activation states.

In the present study, the IPA software found a high number of biological functions that were significantly associated with the proteins in cholesteatoma that were up- or down-regulated and was able to predict activation states in several cases. Table 2 shows these predictions and leaves out all other significantly associated biological functions for which IPA could not estimate the activation states. Comparing cholesteatoma tissue with tympanic membrane and EACS, the following top scoring biological functions with indications of activation direction were identified (Table 2): Decreased adhesion, movement, and differentiation; Increased cell death, generation of reactive oxygen species (ROS), and migration of endothelial cells.

**Table 2:** Activation direction of biological functions in cholesteatoma.

Biological function	Activation	z-score	Comparison	Number of assigned regulated proteins
Infection of cells	decreased	−2.953	C vs T	12
Binding of cells	decreased	−2.707	C vs S	9
Adhesion of tumor cell lines	decreased	−2.377	C vs S	10
Cell movement of epithelial cells	decreased	−1.969	C vs S	4
Cell movement of breast cancer cell lines	decreased	−2.39	C vs S	7
Cell death	increased	+1.751	C vs S	43
Differentiation of epithelial cells	decreased	−1.686	(C & N)* vs S	5
Migration of endothelial cells	increased	+1.65	(C & N)* vs S	8
Generation of reactive oxygen species	increased	+1.951	C vs (T & S)*	4

Statistical predictions on the direction of activation of biological functions associated with cholesteatoma (see experimental procedures). Biological functions with z - scores of the predictions above the numerical value 1.645 (90% significance level) are shown. In a first step, biological functions were statistically associated with cholesteatoma based on the up- and down-regulated proteins that could be assigned to these functions. Subsequently, predictions on the activation direction were calculated from the composition of up- and down-regulations among these proteins. The listed results were found in comparisons between cholesteatoma sack (C), neck of cholesteatoma (N), tympanic membrane (T), and external auditory canal skin (S). *The proteins had to show the same direction of expression in both tissues and meet the fold change criteria in at least one of the tissues.

#### Canonical pathways

**Figure 3:**
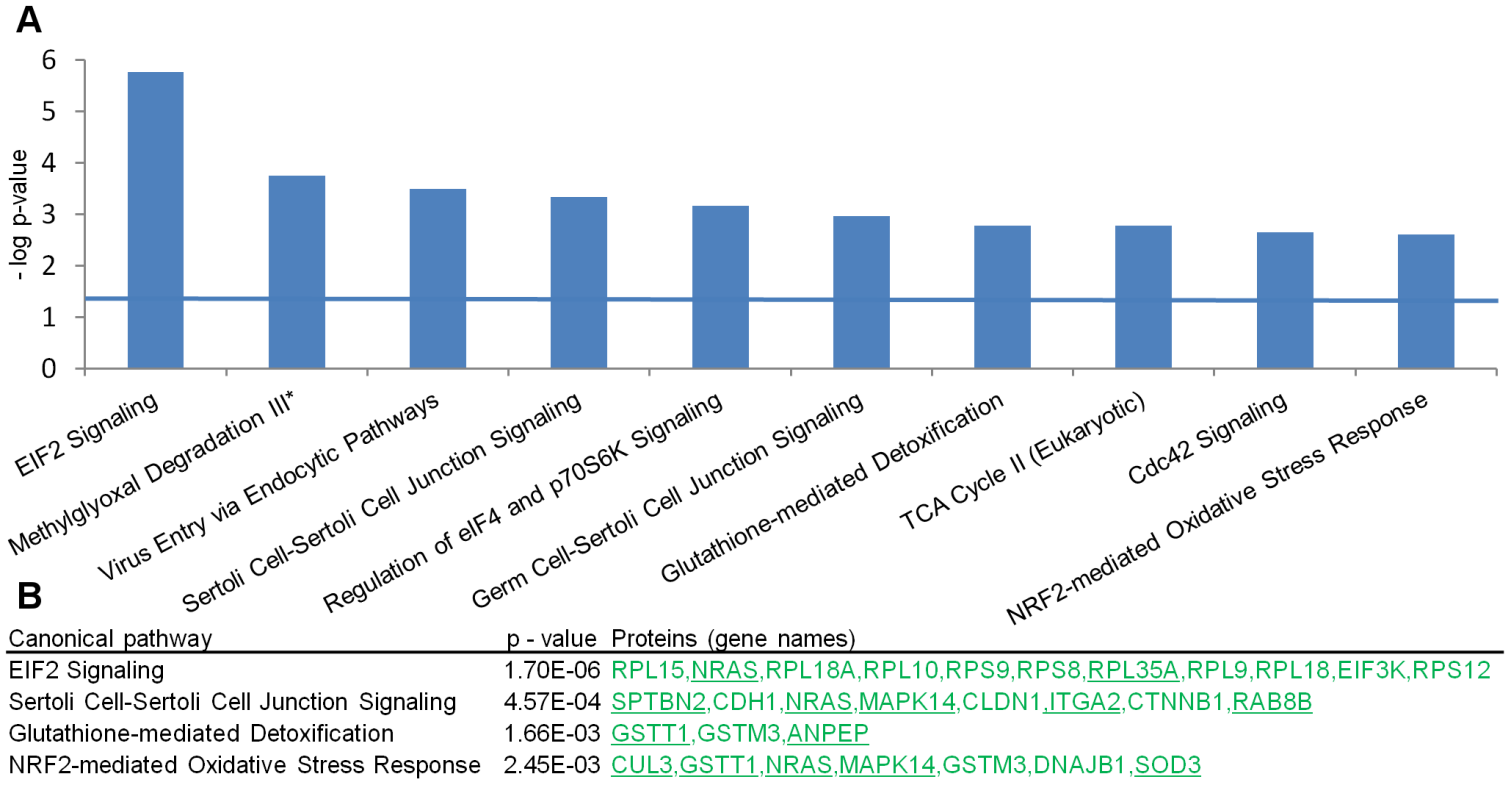
Canonical pathways associated with the proteins meeting the fold-change criteria. A. The top scoring (lowest *p*-values) canonical pathways associated with the differentially expressed proteins found in the pairwise comparisons between the four keratinizing tissues. *The second highest score "Methylglyoxal Degradation III" was found in the comparison between tympanic membrane and EACS. All others were found in the comparison between cholesteatoma and EACS. Horizontal blue line indicates *p* value  = 0.05. B. *p* values and the involved differential-level proteins of three selected pathways from the comparison between cholesteatoma and EACS. All proteins showed lower levels (green color) in cholesteatoma compared with EACS. Underlined proteins: Group B proteins.

**Figure 4:**
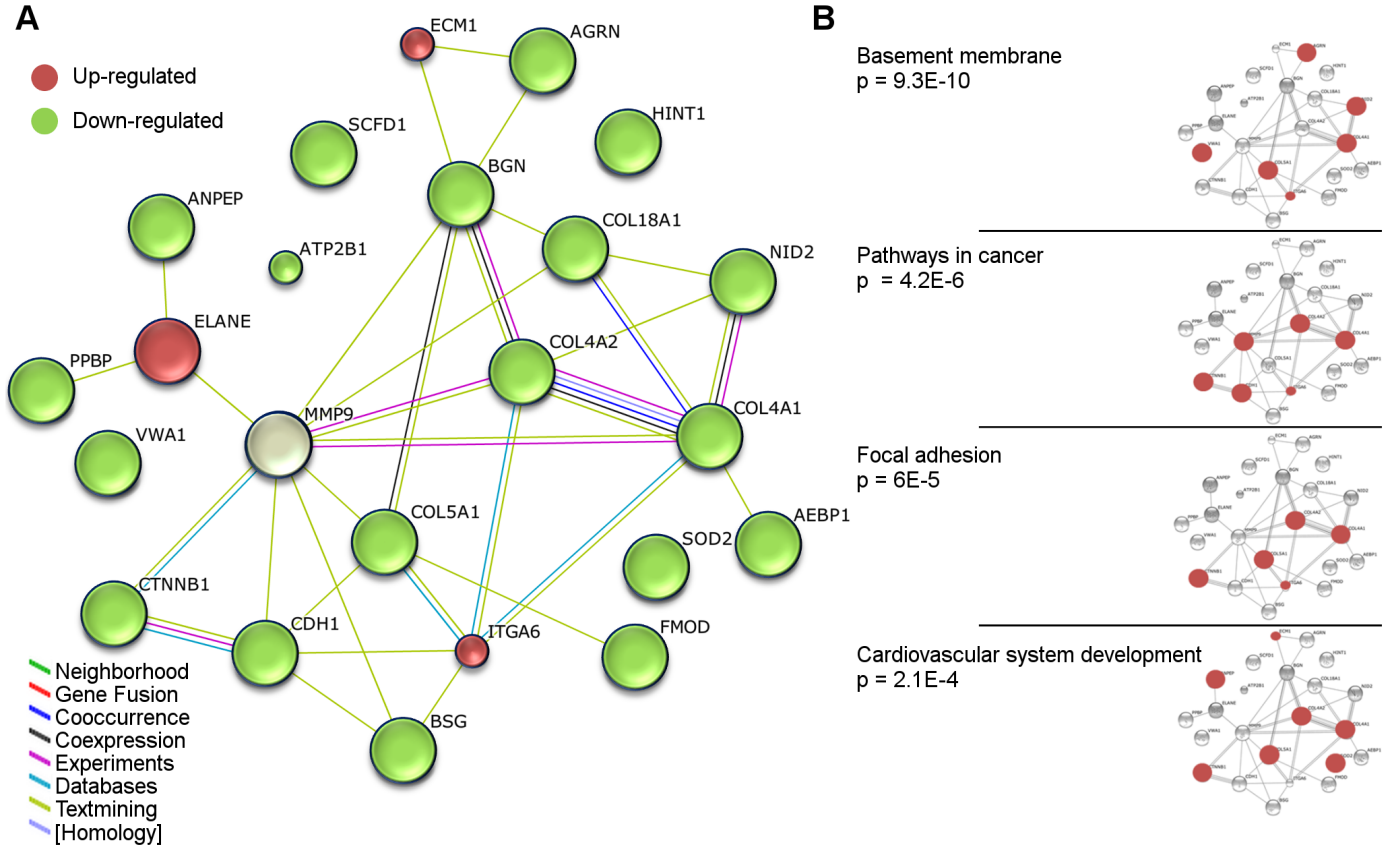
Network of differential level proteins in cholesteatoma with associations to connective tissue. A. The top scoring, automatically-synthesized network of related proteins in IPA: Connective Tissue Development and Function, Embryonic Development, Organ Developmentive tissue.d proteins: Group B proteins.d EACS.holesteatoma a STRING. All proteins, except for MMP9, showed the same expression direction comparing cholesteatoma with tympanic membrane and EACS, respectively. Protein level differences meeting the group A or B criteria were detected in at least one of the two comparisons. 41 interactions (7.95 expected) were identified between the 23 proteins, network *p* value = 1.11e-16. B. Some of the top scoring significant associations of the network with: GO Biological Processes, GO Cellular Components, and KEGG Pathways; ordered by *p* value.

The top scoring canonical pathways from the pairwise tissue comparisons are shown in Figure 3. Nine of the ten highest scoring pathway associations of the differentially expressed proteins were found in tissue comparisons including cholesteatoma indicating that this tissue type is distinct from the others. Along with the “Glutathione-mediated Detoxification” and “Nrf-2 mediated oxidative stress response”, the regulation of the highest scoring pathway “eIF2 Signaling” is likely a response to cellular stress in cholesteatoma tissue. The down-regulated proteins of the “-cell junction signaling” pathways indicate alterations in cell to cell contact and tissue organization as also indicated by the generated network of extracellular matrix related proteins (Figure 4).

#### Networks

Based on information in the Ingenuity Knowledge Base, IPA synthesized the strongest possible networks from the proteins showing differential levels. Synthesized networks helped to visualize the relationships, interactions, and possible consequences of up- and down-regulations of proteins. Networks are often complex and can involve a number of pathways. To enhance subareas of interest, we selected parts of the networks and supplemented them with additional proteins. This was done using Gene Ontology to search for related proteins followed by STRING network analysis.

Figure 4 shows a STRING network that was built from the top scoring synthesized network in IPA mainly consisting of proteins for “connective tissue development and function”. Neutrophil elastase (ELANE) and extracellular matrix protein 1 (ECM1) proteins were up-regulated in cholesteatoma. MMP9 was slightly up-regulated compared with EACS, and clearly down-regulated compared with the tympanic membrane. In contrast, the remaining proteins were all down-regulated. Although these down-regulated proteins are mostly structural proteins, various local actions were also significantly associated, as indicated in the simple functional overlays to the right in Figure 4.

**Figure 5:**
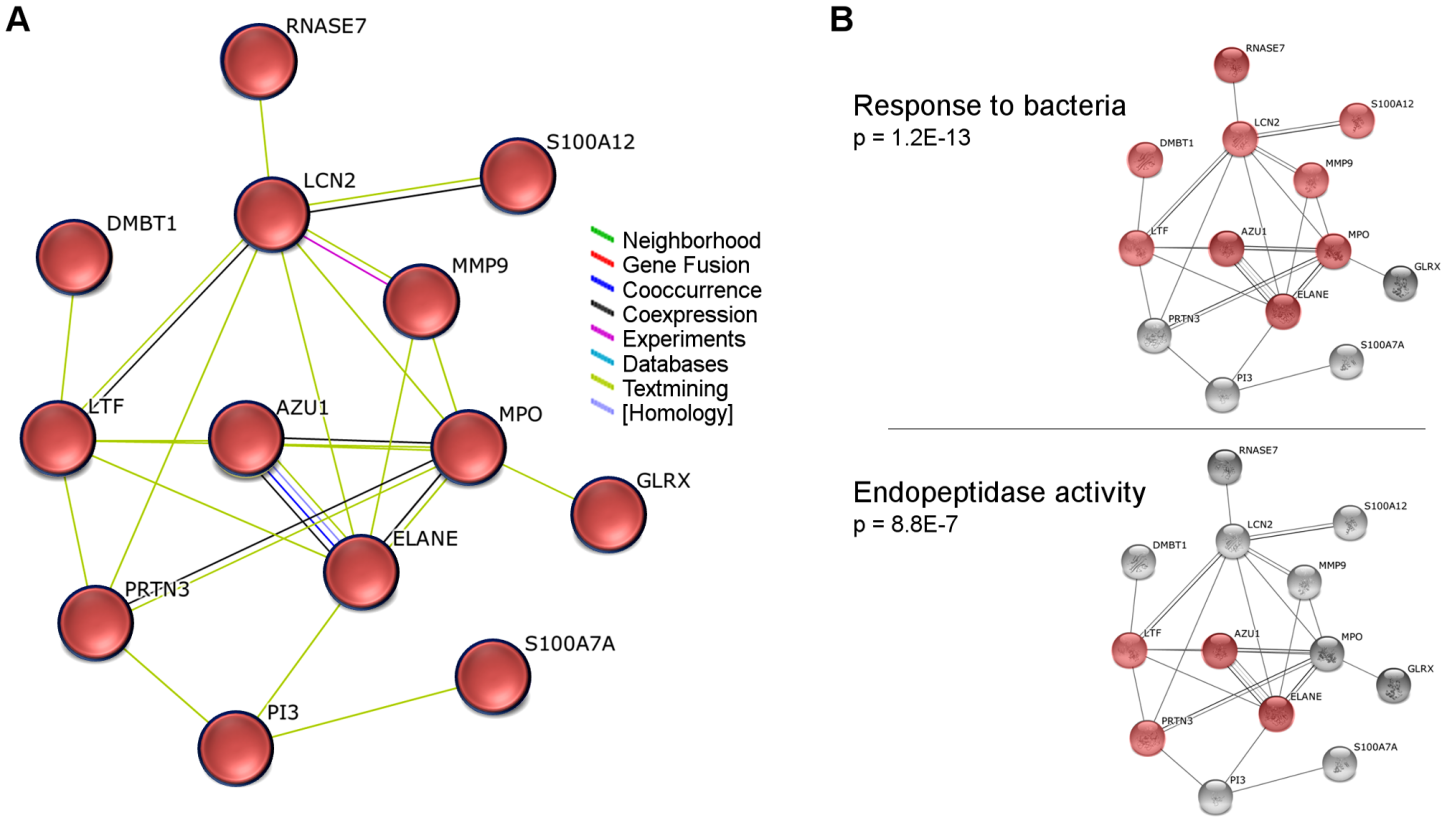
Synthesized network of immune response-related up-regulated proteins in cholesteatoma and neck of cholesteatoma. A. All proteins showed higher protein levels in cholesteatoma and neck of cholesteatoma compared to the external auditory canal skin. The proteins met the fold change criteria in at least one of the two tissue comparisons. B. Examples of significant associations of the network with: GO Biological Processes, GO Cellular Components and KEGG Pathways; ordered by *p* value. The networks were generated in STRING.

**Figure 6:**
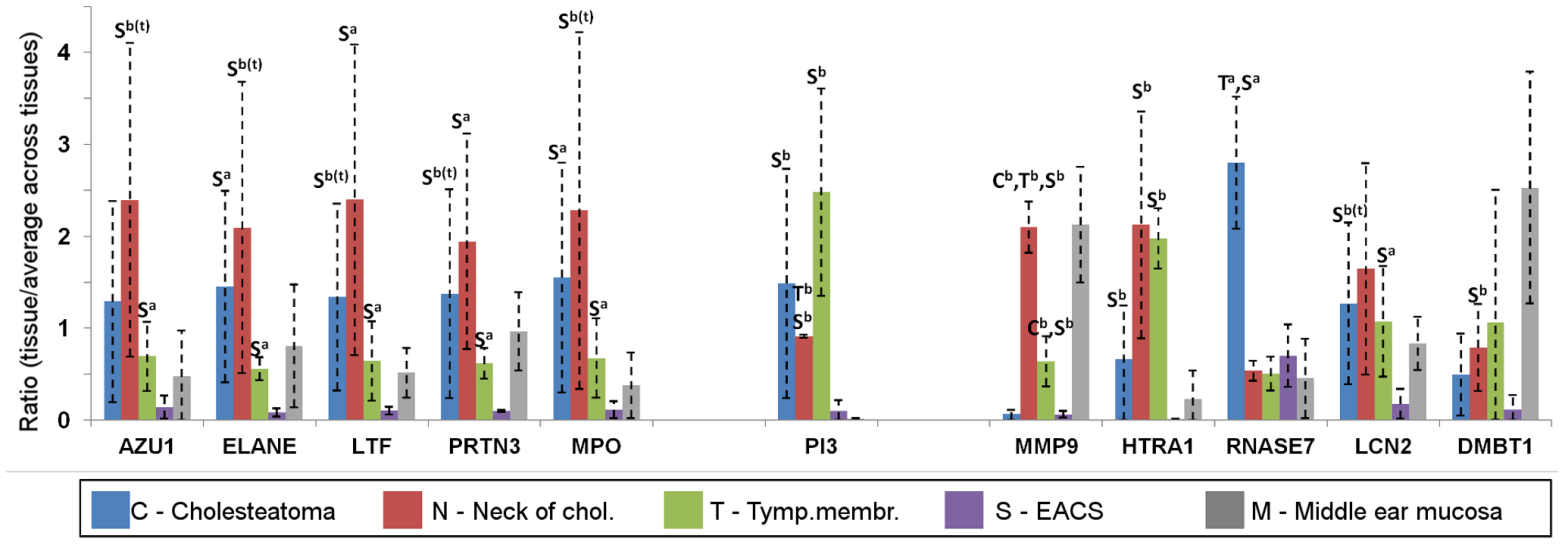
Overview of levels of 11 related proteins involved in inflammation, response to bacteria, and/or protein degradation. Letters above the standard deviation bars indicate the tissues compared to which differences were found in the pairwise comparisons between the four keratinizing tissues. Left: Leukocyte-associated proteins. Middle: Inhibitor of enzyme activity. Right other protein degrading and/or immune-response related proteins. a: Group A proteins, b: Group B proteins, b(t): Triplicate values in group B. Gene names below the columns. Tymp Membr: Tympanic membrane; Chol: Cholesteatoma.

Due to the finding of increased levels of neutrophil elastase (ELANE) in cholesteatoma, we constructed a network of related proteins meeting the criteria for differential protein level in one or both of the comparisons of cholesteatoma vs. EACS or neck of cholesteatoma vs. EACS (Figure 5). All proteins, except RNASE7, showed increased expression in both cholesteatoma and neck of cholesteatoma, and showed significant overlaps with the biological process "response to bacteria" and the molecular function "endopeptidase activity" according to the STRING analysis. High levels of proteins related to ‘response to bacteria' and proteins with protease activity were found, especially in the neck of cholesteatoma (Figure 6); several of these proteins were leukocyte-associated (Figure 6, left). A possible protein breakdown from bacteria induced-inflammation is thereby likely. This can have consequences for the integrity of the tissue.

### Proteins with altered levels in cholesteatoma

Tables 3 and 4 show the top 20 proteins in cholesteatoma compared with neck of cholesteatoma, tympanic membrane, and EACS, according to the sorting described below. Proteins that showed extreme levels in cholesteatoma (compared with any tissue type) and proteins uniquely regulated in cholesteatoma (highly altered levels compared with more than one reference tissue type) were of specific interest. More than one sorting criterion were therefore applied: 1: Number of reference tissues compared to which the same expression direction (up- or down regulation) was found in cholesteatoma, 2: Protein group/fold change criteria (first A then B), and 3: Fold change. On average, approximately four times more proteins were found to have lower levels of expression than higher levels of expression in cholesteatoma compared with the reference tissues (see Table S3). The main findings are the well represented up-regulated active/enzymatic proteins and the down-regulated structural proteins. These regulations may be linked.

**Table 3:** Top 20 Up-regulated proteins in cholesteatoma.

Gene name	Accession #	Protein description	Fold changes of protein levels from the pairwise tissue comparisons					
			C vs N		C vs T		C vs S	
RNASE7	Q9H1E1	Ribonuclease 7	9.59		**6.00**	a	**7.09**	a
KRT4	P19013	Keratin 4	**7.88**	a	3.72		**41.7**	b(t)
S100A7A	Q86SG5	Protein S100-A7A, Koebnerisin	**179**	b(t)	242		**963**	b(t)
CEACAM6	P40199	Carcinoembryonic antigen-related cell adhesion molecule 6	3.81		**14.2**	b	**225**	b
SERPINB10	P48595	Serpin B10	23.0		**6.99**	b	**102**	b
STS	P08842	Steryl-sulfatase	3.40		**45.0**	b	**35.0**	b
APOA2	P02652	Apolipoprotein A-II	**16.6**	b	10.90		**30.0**	b
NPC2	P61916	Epididymal secretory protein E1	4.18		**8.81**	b	**6.82**	b
MPO	P05164	Myeloperoxidase	1.35		1.99		**13.52**	a
KRT7	P08729	Keratin 7	0.99		0.56		**10.39**	a
ELANE	P08246	Neutrophil elastase	1.23		2.19		**9.74**	a
ASAH1	Q13510	Acid ceramidase	2.41		3.14		**4.84**	a
SERPINB12	Q3SYB4	SERPINB12 protein	2.71		**3.89**	a	3.81	
KRT19	P08727	Keratin, type I cytoskeletal 19	1.35		0.73		**2.87**	a
HRNR	Q86YZ3	Hornerin	141		**142**	b(t)	140	
ECM1	Q16610	Extracellular matrix protein 1	2.64		**33.2**	b(t)	10.6	
PRTN3	P24158	Myeloblastin	1.12		2.53		**15.7**	b(t)
LCN2	P80188	Neutrophil gelatinase-associated lipocalin	1.24		1.45		**14.5**	b(t)
LTF	P02788	Lactotransferrin	0.98		1.90		**11.5**	b(t)
KRT8	P05787	Keratin 8	1.63		0.62		**5.92**	b(t)

Up-regulated proteins in cholesteatoma sorted by 1: The number of tissues compared to which cholesteatoma showed higher levels of the protein; 2: Protein group (fold change criteria A then B); 3: Fold change. C: Cholesteatoma; N: Neck of cholesteatoma; T: Tympanic membrane; S: External auditory canal skin; a: Protein group A; b: Protein group B; b(t): Triplicates in protein group B.

**Table 4:** Top 20 Down-regulated proteins in cholesteatoma.

Gene name	Accession #	Protein description	Fold changes of protein levels from the pairwise tissue comparisons					
			C vs N		C vs T		C vs S	
SBDS	Q9Y3A5	Ribosome maturation protein SBDS	**0.083**	b(t)	**0.050**	a	**0.042**	a
SMC1A	Q14683	Structural maintenance of chromosomes protein 1A	**0.027**	b	**0.010**	b	**0.008**	b
SULT1A1	P50225	Sulfotransferase 1A1	**0.112**	b	**0.023**	b	**0.072**	b
PPBP	P02775	Platelet basic protein	**0.080**	b	**0.092**	b	**0.041**	b
COL18A1	P39060	Collagen alpha-1(XVIII) chain	**0.100**	b	**0.064**	b	**0.043**	b
ANPEP	P15144	Aminopeptidase N	**0.060**	b	**0.051**	b	**0.048**	b
PFN2	P35080	Profilin-2	0.392		**0.026**	a	**0.026**	a
FMOD	Q06828	Fibromodulin	**0.157**	a	0.173		**0.088**	a
POLR2E	P19388	DNA-directed RNA polymerases I, II, and III subunit RPABC1	0.598		**0.170**	a	**0.209**	a
HINT1	P49773	Histidine triad nucleotide-binding protein 1	0.303		**0.171**	a	**0.247**	a
CRYAB	P02511	Alpha-crystallin B chain	36.6		**0.312**	a	**0.244**	a
AK2	P54819	Adenylate kinase 2, mitochondrial	1.24		**0.299**	a	**0.378**	a
COCH	O43405	Cochlin	0.134		**0.100**	b(t)	**0.078**	b(t)
VWA1	Q6PCB0	von Willebrand factor A domain-containing protein 1	0.404		**0.116**	b(t)	**0.135**	b(t)
LUC7L2	Q9Y383	Putative RNA-binding protein Luc7-like 2	0.542		**0.007**	b	**0.045**	b
AEBP1	Q8IUX7	Adipocyte enhancer-binding protein 1	0.503		**0.016**	b	**0.016**	b
HIST1H2AB	P04908	Histone H2A type 1-B/E	**0.014**	b	**0.082**	b	0.517	
NID2	Q14112	Nidogen-2, Entactin	0.109		**0.022**	b	**0.016**	b
ABI3BP	Q7Z7G0	Target of Nesh-SH3	**0.112**	b	0.522		**0.021**	b
TNXB	P22105	Tenascin-X	**0.084**	b	0.312		**0.027**	b

Down-regulated proteins in cholesteatoma sorted by 1: The number of tissues compared to which cholesteatoma showed higher levels of the protein; 2: Protein group (fold change criteria A then B); 3: Fold change. C: Cholesteatoma; N: Neck of cholesteatoma; T: Tympanic membrane; S: External auditory canal skin; a: Protein group A; b: Protein group B; b(t): Triplicates in protein group B.

### Keratins

**Figure 7:**
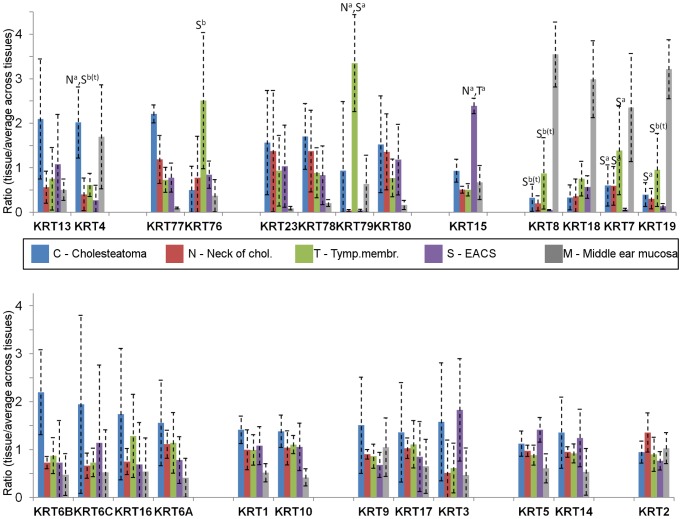
Cytokeratins. All identified cytokeratins (Hair- and hair follicle keratins excluded) were grouped according to Moll *et al*.[38]. Letters above the standard deviation bars indicate the tissues compared to which differences were found in the pairwise comparisons between the four keratinizing tissues (results from comparisons including the middle ear mucosa are not shown). a: Group A proteins; b: Group B proteins; b(t): Triplicate values in group B. Gene names shown below the columns. Tymp Membr: Tympanic membrane; Chol: Cholesteatoma.

The cytokeratin pattern is often used to help describe the level of differentiation of epithelia. Among the five tissue types, cholesteatoma showed a tendency towards the highest levels of cytokeratins (15 out of 25 cytokeratins; Figure 7). Five of these (KRT4, 77, 23, 78, and 80) met the fold change criteria. Four keratins were found to have higher levels of expression compared with middle ear mucosa (KRT77, 23, 78, and 80). One keratin (KRT4) showed a higher level of expression compared with both the neck of cholesteatoma and EACS. Other keratins had increased levels of expression in non-cholesteatoma tissues: KRT76, 79 (Tympanic membrane), KRT15 (EACS), and KRT7, 8, 18, 19 (Mucosa). This latter group comprises keratins of simple epithelia, which are normally expressed in the mucosa but not in the skin [38], and, interestingly, EACS showed lower levels of these proteins compared with both cholesteatoma, neck of cholesteatoma, and especially compared with the tympanic membrane. The increased complexity of the cytokeratin pattern and the up-regulation of KRT4 suggest a lower grade of differentiation of cholesteatoma epithelium compared with the other keratinizing tissues.

### Validation

To test the reproducibility of the protein level differences found between cholesteatoma and EACS, 16 proteins were analyzed by SRM mass spectrometry (Figure S4). The SRM analyses were carried out on another set of patient samples (n≥9) measuring each sample separately (no pooling). A set of proteins that was found to be up-regulated (RNASE7, S100A7A, KRT4, ELANE, and ECM1) or inconsistently up-regulated (S100A7) in cholesteatoma in the large-scale analysis was also found to be significantly up-regulated (p<0.05) in the SRM analyses.

The down-regulations of PFN2, NID2, COL18A1, and GSTM3 were also confirmed by SRM, whereas FMOD escaped detection in SRM. Furthermore, three proteins (SBDS, EIF3K, NRAS) could not be confirmed since they showed high variance and had low magnitude of alterations. Another three proteins (CTNNB1, DNAJB1, S100A16) could be confirmed qualitatively by having clear fold change in line with the large-scale study but with insufficient *p* values (p<0.15). The analysis of single samples, instead of pooled samples, meant that a lower protein amount (approximately 50%) was accessible for SRM analyses compared with the large-scale analyses, and no pre-fractionation was performed. These factors may have contributed to the failure to detect the FMOD protein in the SRM analyses. Out of the 16 tested proteins, 13 showed data matching the large-scale study (for details see Figure S4 and Methods S1).

## Discussion

The identification and relative quantification of more than 2,400 unique proteins and the inclusion of five tissue types allowed for a broad bioinformatics analysis, combining groups of related proteins with differential levels into strong joint estimates. Considering the complexity of tissue biopsies in general and the differences between the five different tissue types, the profiles of the protein extracts showed large overlaps between tissues and a good reproducibility between replicates of the same tissue. Of the proteins identified in all three experiments, on average, 92% were found in all replicates of a given tissue type. A meaningful interpretation of the large amounts of data generated from large-scale proteomics studies requires systematic bioinformatics processing in the context of the disease. The biology most strongly associated with cholesteatoma is discussed in this study.

### Extracellular matrix and basement membrane

The top IPA network synthesized from differentially-regulated proteins in cholesteatoma comprised mainly extracellular matrix-associated components including basement membrane related proteins. One of the components in the hypotheses concerning cholesteatoma pathogenesis is the breach/loss of basement membrane that allows for the invasion of keratinocytes. Sudhoff and colleagues found scattered discontinuities in the staining for collagen IV in cholesteatoma [39]–[41], whereas a continuous collagen IV distribution similar to that of EACS was found in other studies [42]. Shunyu et al did not detect disruptions in the basement membrane of 48 cholesteatomas; however, the middle collagen layer was greatly reduced/lost in most retraction pockets investigated [43]. The fact that only some studies managed to detect these defects suggests that the breaches are either rare and focal, or that they, perhaps instead, are invisible widenings/thinnings of the basement membrane mesh that allow for transmigration of cells. Inclusion/invagination of epithelium with or without basement membrane breach is another possible mechanism for the introduction of epidermal cells to the middle ear [41]. Regardless of the mechanism, changes in the extracellular matrix around the basement membrane reflect consistent findings.

Several differentially-expressed extracellular matrix related proteins were identified in the present study. Indicated by the associated functions in Figure 4 (cancer, adhesion, and cardiovascular system) and by the activated biological functions in Table 2 (e.g. decreased 'Adhesion of tumor cell lines' and increased 'Migration of endothelial cells'), these proteins should not just be regarded as structural parts that form a physical barrier, but as active players that control the environment, and which in this case may allow for angiogenesis and cancer-like reorganization of the tissue. ECM1 was one of the few up-regulated extracellular matrix associated proteins. This protein has previously been found to be up-regulated at the mRNA level in cholesteatoma compared with retroauricular skin [28]. It interacts with many other extracellular matrix proteins, acts as negative regulator of bone mineralization, promotes angiogenesis, and may inhibit MMP9 action [44]. It has also been associated with migration and invasion in cancer [45], and the epidermal expression is minimal under normal conditions.

In the extracellular matrix network (Figure 4) and from the top-scoring proteins in Tables 3 and 4, several other differentially-expressed proteins in cholesteatoma showed regulations that can promote cancer-like alterations. Down-regulation of the nidogens (NID1 and NID2) de-stabilizes the basement membrane and has been associated with cancer formation [46], [47]. Neck of cholesteatoma and cholesteatoma sack showed very low levels of these proteins (NID2 showed more than 20-fold lower levels compared with the tympanic membrane and EACS). The cell adhesion protein CEACAM6 was found to be highly up-regulated in cholesteatoma compared with the tympanic membrane and EACS, in particular. Over-expression has been shown to increase tumor growth and suppress PI3/AKT-dependent apoptosis in head and neck cancer [48]. Its mRNA levels have recently been shown to be up-regulated, as well [29]. Profilin-2 (PFN2) was deficient in the three cholesteatoma replicates, but was found in high levels in all three replicates of the tympanic membrane, EACS, and mucosa. Profilin-2 is a regulator of actin polymerization, and it has recently been shown that its down-regulation enhances invasion of cells [49]and it is associated with poor prognoses in cancer [50].

### Growth of cholesteatoma

Investigations of factors with influence on the growth of cholesteatoma, such as proliferation [13]–[17] and apoptosis [18], [19], have shown varying results. Our recent cytokine analyses revealed an up-regulation of the skin hyperplasia inducing [51] IL-21 in cholesteatoma [52]. The decreased migration of epithelial cells combined with increased cell death indicated by the present study (Table 2) may contribute to the accumulation of material and expansion of the tumor. The special self-cleaning properties of the tympanic membrane and ear canal depend on efficient lateral migration and controlled desquamation, and disturbances in the desquamation of the ear canal skin have previously been shown to halt migration [53].

Accumulation of white greasy scales is one of the most striking characteristics of the macroscopic appearance of cholesteatoma. Steroid sulfatase (STS) is one of the enzymes most strongly associated with desquamation of the skin and is important for the lipid composition and integrity of the skin barrier. Up-regulation, as measured in the present study, can lead to increased detachment of cells and increased desquamation rates, whereas the opposite results in decreased desquamation and thickening of the skin as seen in X-linked ichtyosis [54], [55]. A previous study investigated ear canal skin and found a gradient of STS, with higher levels in the deep medial part compared with the lateral part. The authors speculated that it may play a role in the detachment and migration of cells, and that dysregulation can lead to increased desquamation and accumulation of debris [56]. In the present study, around 30–40 fold higher levels of STS were found in cholesteatoma compared to the tympanic membrane and EACS, whereas a counter-acting enzyme Sulfotransferase 1A1 (SULT1A1) was down-regulated to the same degree, perhaps indicating activation and a resulting over-desquamation.

### Protein-degrading enzymes

Neutrophil elastase (ELANE) was one of two up-regulated proteins in the network of otherwise down-regulated extracellular matrix associated proteins (Figure 4). It is a protease of polymorphnuclear cells that, in addition to its antimicrobial properties, also hydrolyzes a wide range of other proteins, including extracellular matrix proteins like collagen IV [57]; regulation of its activity is important for balance between beneficial and harmful effects. Both cholesteatoma and the neck of cholesteatoma showed higher levels of ELANE compared to EACS, and higher, but more inconsistent levels, compared with the tympanic membrane. In Figures 5 and 6, the neck of cholesteatoma in particular is rich in proteins with the capacity of degrading extracellular matrix (e.g. PRTN3, ELANE, MPO, MMP9, and HTRA1). The significantly associated biological function "response to bacteria" in figure 5 indicates, that bacteria, in this case can evoke an immune response. No bacterial proteins were detected inside the tissues in present study (checked by database searches of MS data against a bacterial database), but the presence of bacteria has previously been detected in other cholesteatoma studies [21], [22]. As bacteria are ubiquitous on epidermis surfaces, their role in cholesteatoma is not clear. A constant presence of bacteria may chronically attract and activate immune cells, which have been found in high numbers in cholesteatoma [58]–[60]. This possible chronic bacteria-induced inflammatory activation of proteases may explain the low abundance of extracellular matrix proteins in cholesteatoma. Among the up-regulated proteases in cholesteatoma tissues, HTRA1 (High temperature requirement A1, serine protease) is a novel finding which has interesting characteristics. This protease has been found in high levels in rheumatoid arthritis, osteoarthritis [61], and Alzheimer's disease [62]. It has a variety of targets, degrades extracellular matrix proteins, and inhibits matrix mineralization and mineral deposition by osteoblasts [63]. HTRA1 may therefore also have a role in the erosion of the ossicles that that is associated with cholesteatoma disease.

### Down-regulation of translation (eif2) and oxidative stress (Nrf2) signaling

“eIF2 signaling” is the canonical pathway that showed the strongest association to the regulated proteins in cholesteatoma. A down-regulation of this pathway, which is a known reaction to cellular stress, reduces the global protein synthesis to limit the detrimental effects of toxins and ROS. All 11 associated proteins from this pathway were down-regulated in the large-scale analysis. However, the down-regulation of two representative proteins, NRAS and EIF3K did not reach significance in the validation analyses, indicating that parts of this stress pathway have high biological variation. The proteins associated with other high scoring stress-related canonical pathways (“Nrf2-mediated oxidative stress response” and “Glutathione-mediated detoxification”) were down-regulated as well. Chronic inflammatory cellular stress, as seen in cholesteatoma, is associated with a down-regulation of the Nrf2 pathway [64], [65], which in turn leads to reduced transcription of glutathione metabolism related proteins [66]; the result is increased sensitivity to stress followed by increased cell death. Two recent studies measured a significantly higher total oxidant and significantly lower total antioxidant status in serum of patients with cholesteatoma [67], [68]. It is possible that increased production of ROS in cholesteatoma (as indicated in Table 2) leads to disproportionate extracellular matrix damage due to impaired defense against stress.

### Cytokeratins and differentiation

Cytokeratin profiles can help assess the level of differentiation of epithelia. KRT1, 4, 5, 6, 7, 8, 10, 13, 14, 15, 16, 17, 18 and 19 have previously been detected in human cholesteatoma tissue [6]–[11], [29]. All investigators (except Klenke *et al*.[29]) used antibody-based methods but varied in other parameters studied, such as the type of reference tissue, which may partly explain the inconsistencies between the studies. The present study replicated the identification of all the above-mentioned cytokeratins. In addition, the cytokeratins 2, 3, 9, 23, 76, 77, 78, 79, and 80 were also identified (excluding 11 identified hair/hair follicle keratins). All cytokeratins were identified in all five tissues. KRT4 is a keratin of mucosal stratified squamous epithelia and is normally absent in epidermis [38]. In the present study, it was detected in all tissues and found in higher levels in cholesteatoma compared to the neck of cholesteatoma and EACS, indicating alterations and poorer differentiation of the cholesteatoma keratinocytes. The higher levels of the primary- and secondary cytokeratins of simple epithelial cells (KRT 7, 8, and 19) in cholesteatoma and/or the tympanic membrane in comparison with EACS also support this finding. Vennix *et al*. found induction of KRT4 and KRT7 in a meatal-to-middle ear skin graft animal model [4]. KRT18 and 19 mRNA have also been found to be increased in cholesteatoma compared with retroauricular skin [28] and EACS [29]. In the present study, there was a tendency towards higher levels of a wide range of cytokeratins in cholesteatoma (15 out of 25) compared with the other keratinizing tissues. Increased complexity of the cytokeratin expression pattern often parallels the reduction in the degree of differentiation found in reactive conditions [38]. Both the general tendency and the specific up-regulated cytokeratins in cholesteatoma in this study indicate a lower degree of differentiation compared with the tissues from which it is believed to originate, which could have big consequences on the behavior of the cells in cholesteatoma.

### S100 proteins

Multiple S100 proteins showed high levels of expression in cholesteatoma. In the large-scale analysis, Hornerin (S100A16/18), Koebnerisin (S100A7A), Calcitermin (S100A12); and (in two out of three ratios) Psoriasin (S100A7), Calgranulin A (S100A8), and Calgranulin B (S100A9) were all found in very high levels in cholesteatoma tissue. S100 proteins have previously been found to be up-regulated at the protein level (S100A8, S100A9) [69], and at the mRNA level (S100A7, S100A8, S100A9, S100A7A and S100A12) [27], [29], [30], [70] in cholesteatoma. Hornerin expression is a novel finding, and in the large-scale analysis, it showed around 150 times higher levels in cholesteatoma compared to the other tissues. Up-regulation was also found in the SRM analysis but with high variation (*p* = 0.13). Hornerin is a part of the cornified envelope in the stratum corneum, and may play a role in the barrier functions of the skin. It has been observed in high levels in psoriasis and in healing wounds [71]. Increased levels of S100A12 have been significantly associated with increased cell death, tissue damage, and thoracic aortic aneurysm [72]. Psoriasin and Koebnerisin both work as "alarmins" that amplify the inflammatory response through the induction of cytokine production from keratinocytes [73], and have therefore been proposed as targets in inflammatory diseases [74]. A study that compared the expression of S100A1, 2, 3, 4, 5, 6, and S100B in different epithelial lesions in the head and neck found these proteins to be most frequently expressed in craniopharyngeomas and cholesteatomas [75]. Several S100 proteins are expressed in the epidermis, and many are highly overexpressed in a number of pathological conditions, such as skin barrier dysfunction, wound healing, psoriasis, cancer, cellular stress, inflammation, and infection [76]–[79]. Having common but also distinct biological functions, their roles in cholesteatoma disease may be diverse. The potent pro-inflammatory capacity of these highly up-regulated proteins however, makes a central role in the pathogenesis of cholesteatoma likely.

### Design considerations

Proteomics studies require high amounts of protein, which partly explains the low number of cholesteatoma proteomics investigations and the consistent choice of retroauricular skin as reference tissue instead of ear canal skin or membrane [80]–[82]. The methods used in previous studies (2DE - MALDI ToF) are very different from the large-scale proteomics methods used in the present study exemplified by the difference in number of identified proteins (<20 in earlier studies vs >2400 in the present study). The previous 2DE studies share none of the identified proteins, and the small datasets describe no common biological traits for the disease [80]–[82]. An issue that has not often been specified in other studies is the frequent use of topical steroids. Steroid use was not allowed within two weeks prior to biopsy in the present study to avoid an iatrogenic depression of inflammatory mediators, which are of great interest and relevance to cholesteatoma pathology. In addition to a general masking of the inflammatory response, the differences in drug availability of EACS, tympanic membrane, and cholesteatoma could skew the relative levels between the tissues and overestimate the inflammatory action in cholesteatoma. To produce widely applicable protein profiles, patients were not sub-grouped by e.g. age or extent of bone erosion. The degree of inflammation varies over time in cholesteatoma. We consider times of severe infection/inflammation unsuitable for the investigation of the baseline protein expression of cholesteatoma tissue, if such exists; therefore we selected patients that showed no signs of acute inflammation. Retroauricular skin and EACS are very different, and the relative levels of given proteins in cholesteatoma will therefore differ depending on the reference tissue. Most studies have only used one reference tissue, and EACS is the most commonly used. The use of a panel of reference tissues provided a more nuanced picture of the differential protein levels and identified regulations of proteins specific for cholesteatoma.

## Conclusions

This discovery proteomics study implicated several altered biological processes related to the cholesteatoma pathology. From the macroscopic appearance of the tissue, it is not surprising that proteins related to extracellular matrix and basement membrane were prevalent among the regulated proteins in cholesteatoma. The observed down-regulation of several extracellular matrix and basement membrane proteins, such as COL18A1 and NID2, could have great consequences on the integrity of the tissue and lead to altered differentiation (KRT4) and cancer-like alterations that may explain the characteristic phenotype. Up-regulation of proteases, such as ELANE and of pro-inflammatory S100 proteins (e.g. S100A7A and S100A7) were other clear and pronounced protein changes; these regulated biological areas in cholesteatoma may be linked components of a bacteria induced disease mechanism and hold potential as future drug targets.

## Supporting Information

Figure S1 **SDS separation/prefractionation of pooled tissue samples.**
(DOCX)

Figure S2 **Cluster analysis based on the protein levels of 1738 proteins.**
(DOCX)

Figure S3 **Pie charts of the ranges of biological processes in the five tissue types.**
(DOCX)

Figure S4 **Validation of protein alterations in cholesteatoma versus EACS.**
(DOCX)

Table S1 **Number of proteins meeting the group A and B criteria for fold change.**
(DOCX)

Table S2 **Quantitative proteomics data for proteins passing the criteria for fold change (protein groups A and B).**
(XLSX)

Table S3 **Quantitative proteomics data from human middle ear biopsies to study cholesteatoma biology.**
(XLSX)

Methods S1 (DOCX)
