# Supplementary material for: Large-Scale Proteomics Differentiates Cholesteatoma from Surrounding Tissues and Identifies Novel Proteins Related to the Pathogenesis
Source: PLoS One. 2014 Aug 5;9(8):e104103. doi: 10.1371/journal.pone.0104103 (PMC4122447; doi:10.1371/journal.pone.0104103)
Supplement: Figure S1 — SDS separation/prefractionation of pooled tissue samples. (DOCX) [file pone.0104103.s001.docx]

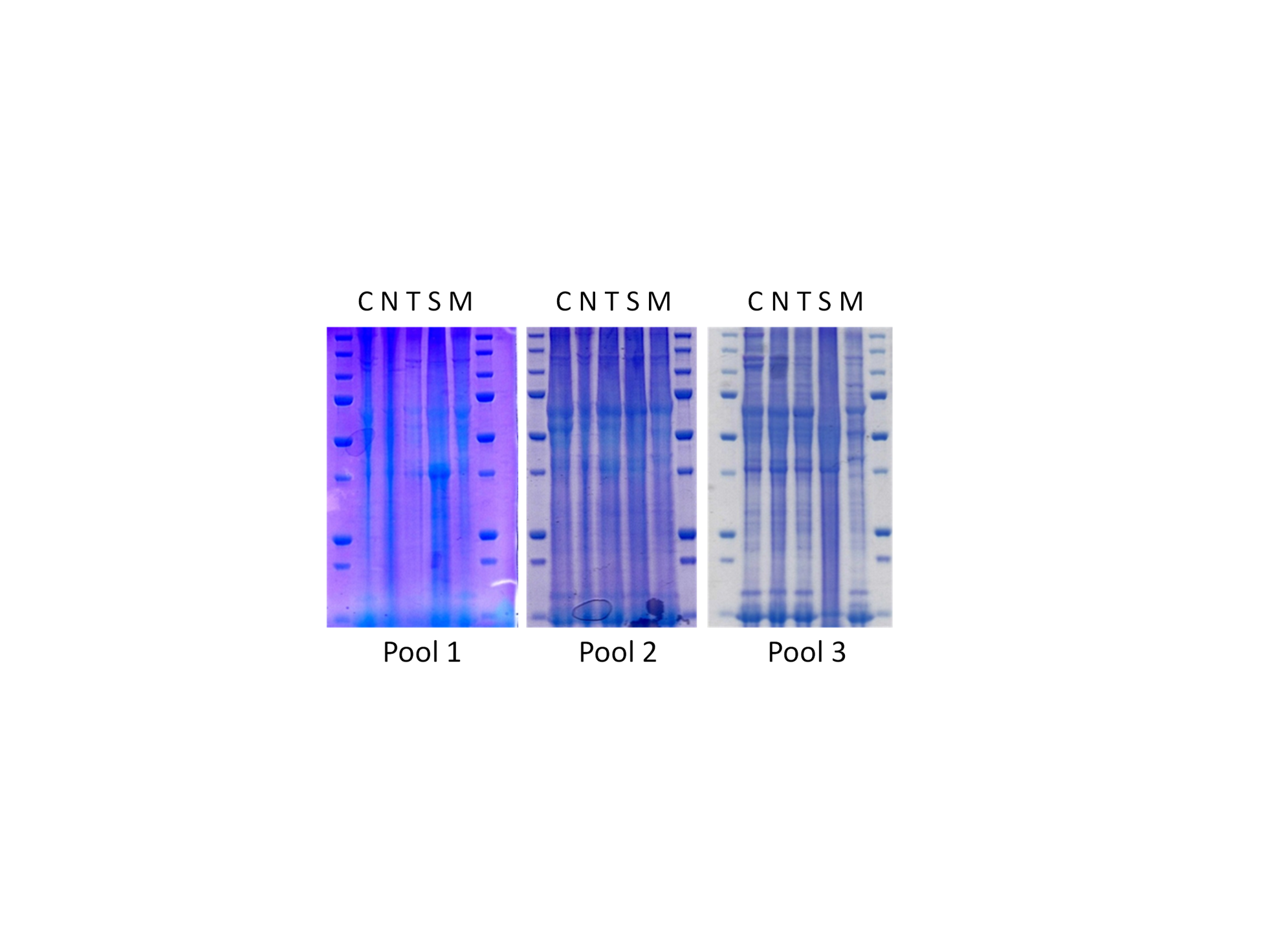


**Figure S1. SDS separation/prefractionation of pooled tissue samples.**

The different tissue sample types within the each pool were run in the same SDS gel prior to prefractionation for LC/MS analyses. C: Cholesteatoma; N: Neck of cholesteatoma; T: Tympanic membrane; S: EACS; M: Middle ear mucosa.
