# Supplementary material for: Large-Scale Proteomics Differentiates Cholesteatoma from Surrounding Tissues and Identifies Novel Proteins Related to the Pathogenesis
Source: PLoS One. 2014 Aug 5;9(8):e104103. doi: 10.1371/journal.pone.0104103 (PMC4122447; doi:10.1371/journal.pone.0104103)
Supplement: Figure S2 — Cluster analysis based on the protein levels of 1738 proteins. (DOCX) [file pone.0104103.s002.docx]

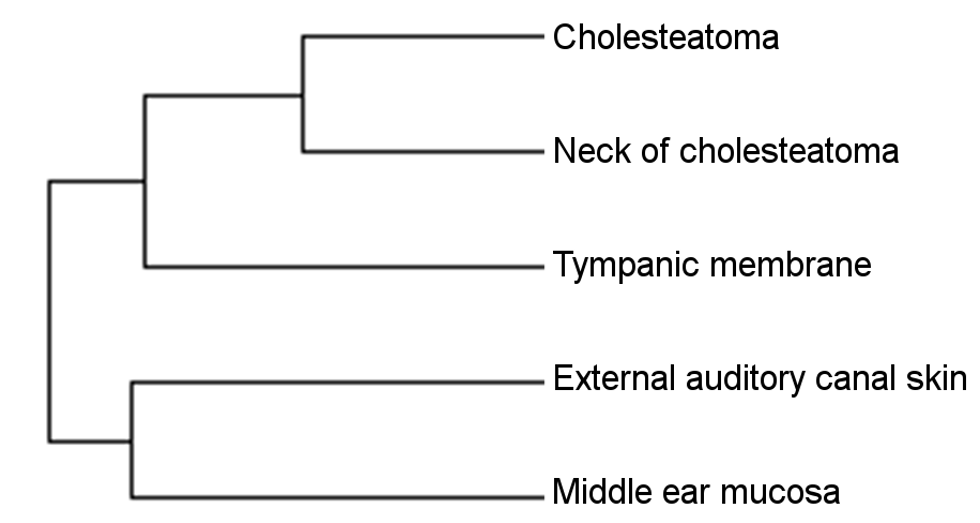


**Figure S2. Cluster analysis based on the protein levels of 1738 proteins.**

The length of the branches and order of the tissues indicate the degree of correlation between the different tissues. The expression profiles of cholesteatoma and the neck of cholesteatoma showed closest relationship (were most similar).
