# Supplementary material for: Large-Scale Proteomics Differentiates Cholesteatoma from Surrounding Tissues and Identifies Novel Proteins Related to the Pathogenesis
Source: PLoS One. 2014 Aug 5;9(8):e104103. doi: 10.1371/journal.pone.0104103 (PMC4122447; doi:10.1371/journal.pone.0104103)
Supplement: Figure S3 — Pie charts of the ranges of biological processes in the five tissue types. (DOCX) [file pone.0104103.s003.docx]

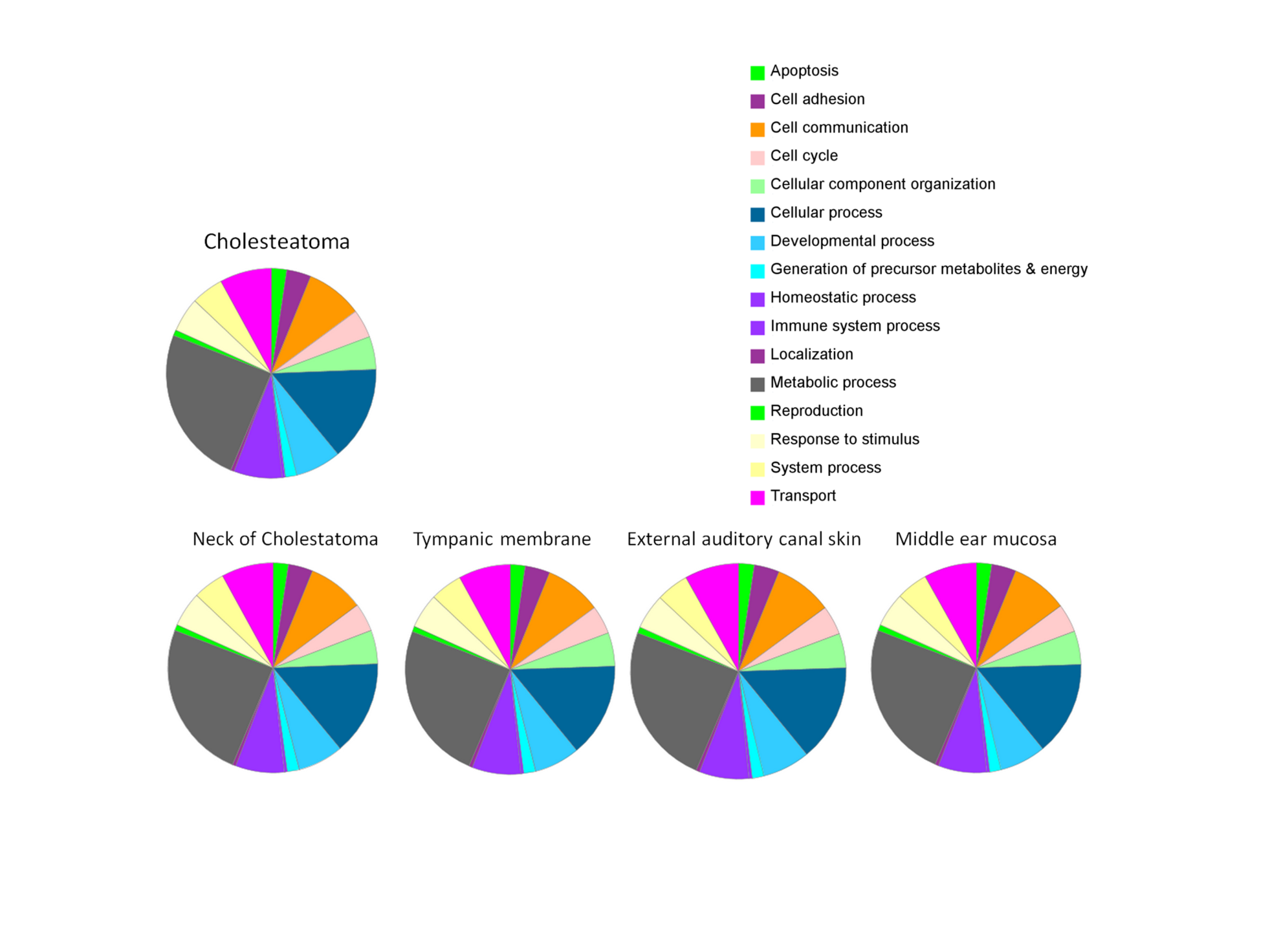


**Figure S3. Pie charts of the ranges of biological processes in the five tissue types.**

Analyses of the protein lists to generate the pie charts were performed in PANTHER (<http://www.pantherdb.org>).
