# Supplementary material for: Large-Scale Proteomics Differentiates Cholesteatoma from Surrounding Tissues and Identifies Novel Proteins Related to the Pathogenesis
Source: PLoS One. 2014 Aug 5;9(8):e104103. doi: 10.1371/journal.pone.0104103 (PMC4122447; doi:10.1371/journal.pone.0104103)
Supplement: Figure S4 — Validation of protein alterations in cholesteatoma versus EACS. (DOCX) [file pone.0104103.s004.docx]

**

**Figure S4. Validation of protein alterations in cholesteatoma versus EACS.**

Selected Reaction Monitoring (SRM) mass spectrometry was applied to validate the data from the large-scale proteomics study. The logarithm of the fold change difference between cholesteatoma and EACS is depicted and error bars correspond to the standard error of the mean. Significantly increased levels in cholesteatoma compared with EACS is annotated as *, ** and *** for *p*<0.05, *p*<0.01 and *p*<0.001, respectively. **A:** Four proteins in ten patient samples (n=10) were successfully validated by SRM on Triple Q MS. On absolute scale, the median protein fold changes were 6.0, 14.0, 3.1, and 3.5 for RNAS7, S100A7, S100A7A, and ELANE proteins, respectively. **B:** Targeted Selected Reaction Monitoring (T-SRM) was applied on Q-Exactive Orbitrap MS to validate 12 additional proteins (in 9 patients). The fold changes and *p* values were: PFN2: down 10.6, *p*=0.005; NID2: down 8.9, *p*=0.010; COL18A1: down 7.4, *p*=0.011; CTNNB1: down 3.8, *p*=0.07; GSTM3: down 3.5, *p*=0.03; DNAJB1: down 2.9, *p*=0.13; SBDS: 1.0, *p*=0.39; EIF3K: 1.2, *p*=0.35; NRAS: 1.3, *p*=0.35; S100A16: up 2.6, *p*=0.13; KRT4: Up 2.6, *p*=0.002; ECM1: up 7.2, *p*=0.001.
