# Supplementary material for: Large-Scale Proteomics Differentiates Cholesteatoma from Surrounding Tissues and Identifies Novel Proteins Related to the Pathogenesis
Source: PLoS One. 2014 Aug 5;9(8):e104103. doi: 10.1371/journal.pone.0104103 (PMC4122447; doi:10.1371/journal.pone.0104103)
Supplement: Table S1 — Number of proteins meeting the group A and B criteria for fold change. (DOCX) [file pone.0104103.s005.docx]

| **Table S1**. **Number of proteins meeting the group A and B criteria for fold change.** | | | | | |
| --- | --- | --- | --- | --- | --- |
| Cholsteatoma vs. | | Neck | Tymp | EACS | Mucosa |
| Group A proteins | | 2 | 18 | 57 | 54 |
| Group B proteins | | 24 | 55 | 102 | 99 |
| Total |  | 26 | 73 | 159 | 153 |
| Only comparisons including cholesteatoma are shown. Only proteins that did not pass the group A criteria were tested by the group B criteria. Neck: Neck of cholesteatoma; Tymp: Tympanic membrane; EACS: External auditory canal skin; Mucosa: Middle ear mucosa. | | | | | |
