# Supplementary material for: Large-Scale Proteomics Differentiates Cholesteatoma from Surrounding Tissues and Identifies Novel Proteins Related to the Pathogenesis
Source: PLoS One. 2014 Aug 5;9(8):e104103. doi: 10.1371/journal.pone.0104103 (PMC4122447; doi:10.1371/journal.pone.0104103)
Supplement: Methods S1 — (DOCX) [file pone.0104103.s008.docx]

**Supporting Materials and methods S1.**

**Gel-based sample pre-fractionation and peptide preparation.**

GeLC-MS/MS methodology was performed as previously described (Hansen et al. e26634). Protein samples, 30 µg of each, were separated by SDS-PAGE in precast Criterion XT Bis-Tris gels, MOPS buffer (BioRad) by applying 200 V until dye front reached the bottom of the gel. The five different tissues within a pool were run alongside in the same gel (one gel per pool). The gel lane of each sample was split into 10 fractions that were cut into 1 mm^2^ cubes. After destaining and reduction/alkylation (TCEP 50 mM/Iodoacetamide 50 mM), the protein was digested by trypsin 0.01 µg/µl overnight at 37 °C. After extraction of the peptides with acetonitrile, the extract was vacuum centrifuged and the peptides were purified using C-18 Pep Clean spin columns (PIERCE) according to the manufacturer’s protocol.

**Selection Reaction Monitoring (SRM) MS assay development**

Based on the large-scale study, proteins were selected for validation by Selected Reaction Monitoring (SRM). Peptide selection as well as method development, method optimization, and data analysis were performed using the open source software Skyline (Ref: doi:10.1093/bioinformatics/btq054). Proteins selected for SRM validation were imported to Skyline and tryptic *in silico* digestions were carried out. Peptide candidates were further selected based on a series of selection criteria. The first peptide requirement was uniqueness in the whole human proteome (HUMAN, Uniprot, reviewed, Ref: doi:10.1093/database/bar009). Secondly peptides were selected based on presence in spectral library databases. Spectral libraries were generated from in-house data from the present study or from the publicly available NIST database (National Institute of Standards and Technology, http://peptide.nist.gov/). Peptides with higher abundance were selected if they in addition showed multiple product ions, of the y-series, in the spectral libraries. Using a built-in filter feature in the Skyline software, the five most intense transitions were selected for each peptide. Synthetic, crude, isotopically labeled analogues for each of the selected peptides were purchased (from JPT, Germany). These tryptic peptides were heavy labeled at the amino acid residues Lysine (+8 Da) and Arginine (+10 Da). Peptides with cysteine residues were purchased as carbamidomethylated. Heavy labeled peptide analogues that were not detectable at concentration level 15 nmole/L were deleted from the method. Furthermore, during method development and optimization the numbers of protein specific peptides were adjusted to one to three peptides as not all of the initially included peptides showed good chromatography as well as sensitivity on the LC-MS. Retention times for all peptides were determined and used to prepare the scheduled SRM-method with SRM time-windows of 4 minutes.

**The following protein specific peptides were analyzed by Triple Q SRM assay:**

**sp|Q9H1E1|RNAS7_HUMAN** *Ribonuclease 7 OS=Homo sapiens GN=RNASE7 PE=1 SV=2*DLNTFLHEPFSSVAAT**C**QTP**K** (charge state +++, product ions y8+, y7+, y6+, y5+, y3+)
DSQQFHLVPVHLD**R** (charge state +++, product ions y6+, y10++,  y9++, y6++, y4++)

**sp|Q86SG5|S1A7A_HUMAN** *Protein S100-A7A OS=Homo sapiens GN=S100A7A PE=2 SV=3*GIHYLATVFE**K** (charge state ++, product ions y9+, y8+, y7+, y6+, y5+) also (charge state +++
product ions y6+, y5+, y4+, y6++, y5++)

**sp|P31151|S10A7_HUMAN** *Protein S100-A7 OS=Homo sapiens GN=S100A7 PE=1 SV=4*GTNYLADVFE**K** (charge state ++, y8+, y7+, y6+, y3+, y9++)

**sp|P08246|ELNE_HUMAN** *Neutrophil elastase OS=Homo sapiens GN=ELANE PE=1 SV=1*VVLGAHNLS**R** (charge state++, product ions y8+, y7+, y5+,y8++, y7++)
LGNGVQ**C**LAMGWGLLG**R** (charge state++, product ions y12+, y9+, y8+, y7+, y5+)
SNV**C**TLV**R** (charge state ++, product ions y6+, y5+, y4+, y2+, y1+)

**The following protein specific peptides were analyzed by Q-Exactive Plus t-SRM assay**

**sp|P39060|COIA1_HUMAN** *Collagen alpha-1(XVIII) chain OS=Homo sapiens GN=COL18A1*

AVGLAGTF**R**

LQDLYSIV**R**

**sp|P35222|CTNB1_HUMAN** *Catenin beta-1 OS=Homo sapiens GN=CTNNB1*

LLNDEDQVVVN**K**

LSVELTSSLF**R**

**sp|P25685|DNJB1_HUMAN** *DnaJ homolog subfamily B member 1 OS=Homo sapiens GN=DNAJB1*

DGSDVIYPA**R**

EIAEAYDVLSDP**R**

**sp|Q16610|ECM1_HUMAN** *Extracellular matrix protein 1 OS=Homo sapiens GN=ECM1*

LTFINDLCGP**R**

**sp|Q9UBQ5|EIF3K_HUMAN** *Eukaryotic translation initiation factor 3 subunit K OS=Homo sapiens* GN=EIF3K

ALTNLPHTDFTLC**K**

ENAYDLEANLAVL**K**

YNPENLATLE**R**

**sp|P21266|GSTM3_HUMAN** *Glutathione S-transferase Mu 3 OS=Homo sapiens GN=GSTM3*

FSWFAGE**K**

IAAYLQSDQFC**K**

YTCGEAPDYD**R**

**sp|P19013|K2C4_HUMAN** *Keratin, type II cytoskeletal 4 OS=Homo sapiens GN=KRT4*

VDSLNDEINFL**K**

VQFLEQQN**K**

**sp|Q14112|NID2_HUMAN** *Nidogen-2 OS=Homo sapiens GN=NID2*

FAVTNQIGPV**K**

VFALYNDEE**R**

**sp|P35080|PROF2_HUMAN** *Profilin-2 OS=Homo sapiens GN=PFN2*

EGFFTNGLTLGA**K**
SQGGEPTYNVAVG**R**

**sp|P01111|RASN_HUMAN** *GTPase NRas OS=Homo sapiens GN=NRAS*

QGVEDAFYTLV**R**

**sp|Q96FQ6|S10AG_HUMAN** *Protein S100-A16 OS=Homo sapiens GN=S100A16*

AVIVLVENFY**K**

**sp|Q9Y3A5|SBDS_HUMAN** *Ribosome maturation protein SBDS OS=Homo sapiens GN=SBDS PE=1 SV=4*LTNVAVV**R**

DIATIVAD**K**

**Sample preparation and LC-SRM-MS analysis**

Peptides were prepared from tissues samples, as described in the large-scale experiment, and 20 µg peptides were dissolved in 35uL buffer A to which approximately 15 nmole/L of each of the heavy labeled peptide standards had been added. Sample injection volume was 14 µL, and pure buffer A was injected between samples to ensure minimal sample carry-over.

The LC-MS system consisted of a Proxeon EASY nano-LC (Proxeon, Odense, Denmark) coupled to a mass spectrometer; either TSQ-Vantage triple quadrupole mass spectrometer (Thermo Fisher Scientific, Waltham, MA, USA) or Q-Exactive Plus (Thermo Fisher Scientific, Bremen, Germany).The LC was operated as C18 based reverse phase separation with a 2 cm trap column (5 µm, ID 100 µm) and a 10 cm analytical column (3 µm, ID 75 µm) (EASY column, Thermo). The columns were mounted in a Thermo Scientific Nanospray Flex Ion Source with liquid junction using a steel emitter. Trap column and analytical columns were equilibrated with 100 % buffer A prior to each analysis. Eluents used were buffer A (H_2_O, 2% acetonitrile, 0.1% HCOOH) and buffer B (Acetonitrile, 5% H_2_O, 0.1% HCOOH). Peptides were eluted from the column using the following gradient. Linear 0- 39% B in 40 minutes, linear 39- 100% in 5 minutes followed by isocratic 100% B 45- 53 minutes. For MS ionization a spray voltage of 1700 V was applied, and all peptides were monitored in the positive ionization mode. On TSQ-Vantage, capillary temperature was set to 200°C and selectivity for both Q1 and Q3 was 0.7 (FWHM). The collision energy for each of the transitions was calculated using Skyline (V 1.4.0.4421). A total of 220 transitions with a cycle time of 1.9 seconds were used in the scheduled SRM method monitoring both the heavy and light peptides. On Q-Exactive Plus the capillary temperature was 250°C and it was operated in targeted MS2 mode with resolution 17,500, normalized collision energy of 27, and automatic gain control target of 200,000 ions.

Raw files were imported to Skyline and each file was quality checked by visual inspection of retention time, integration, and ranking of transitions for each peptide.

**Bioinformatics – canonical pathways and biological functions**

Right-tailed fisher’s exact tests were used to calculate *p* values from two-way tables determining the probability that each canonical pathway or biological function assigned to the proteins with differentially altered levels was due to chance alone. In addition to assessing the strength of these associations, when the data allowed for it, estimates on the activation states of the significantly associated biological functions were stated as well. Z-scores determined whether a biological function had significantly more "increased" than "decreased" predictions, andscores exceeding the numerical value 2 were considered significant.

Reference List

Hansen, J., et al. "Quantitative proteomics reveals cellular targets of celastrol." PLoS.One. 6.10 (2011): e26634.
